# Identification of a novel temperature sensitive promoter in cho cells

**DOI:** 10.1186/1472-6750-11-51

**Published:** 2011-05-12

**Authors:** Haruthai Thaisuchat, Martina Baumann, Jens Pontiller, Friedemann Hesse, Wolfgang Ernst

**Affiliations:** 1 Department of Biotechnology, University of Natural Resources and Life Sciences Vienna, Muthgasse 11, 1190 Vienna, Austria; 2 Austrian Center of Biopharmaceutical Technology, Muthgasse 18, 1190 Vienna, Austria; 3 Austrian Center for Industrial Biotechnology (ACIB), A-8010 Graz, Austria

## Abstract

**Background:**

The Chinese hamster ovary (CHO) expression system is the leading production platform for manufacturing biopharmaceuticals for the treatment of numerous human diseases. Efforts to optimize the production process also include the genetic construct encoding the therapeutic gene. Here we report about the successful identification of an endogenous highly active gene promoter obtained from CHO cells which shows conditionally inducible gene expression at reduced temperature.

**Results:**

Based on CHO microarray expression data abundantly transcribed genes were selected as potential promoter candidates. The *S100a6 *(calcyclin) and its flanking regions were identified from a genomic CHO-K1 lambda-phage library. Computational analyses showed a predicted TSS, a TATA-box and several TFBSs within the 1.5 kb region upstream the ATG start signal. Various constructs were investigated for promoter activity at 37°C and 33°C in transient luciferase reporter gene assays. Most constructs showed expression levels even higher than the SV40 control and on average a more than two-fold increase at lower temperature. We identified the core promoter sequence (222 bp) comprising two SP1 sites and could show a further increase in activity by duplication of this minimal sequence.

**Conclusions:**

This novel CHO promoter permits conditionally high-level gene expression. Upon a shift to 33°C, a two to three-fold increase of basal productivity (already higher than SV40 promoter) is achieved. This property is of particular advantage for a process with reduced expression during initial cell growth followed by the production phase at low temperature with a boost in expression. Additionally, production of toxic proteins becomes feasible, since cell metabolism and gene expression do not directly interfere. The CHO S100a6 promoter can be characterized as cold-shock responsive with the potential for improving process performance of mammalian expression systems.

## Background

The number of recombinant proteins used for therapeutic applications has greatly increased in recent years. For manufacturing complex biopharmaceuticals the Chinese hamster ovary (CHO) expression system is the leading production platform due to appropriate product secretion, post-translational processing, a simple means of cultivation and up-scaling, a reasonable safety profile and last but not least the approval from regulatory authorities [1,3]. To keep pace with the needs of the market, optimizing efforts are required, including also upstream process development. In this context, the genetic vector construct has to be considered since it significantly affects productivity [4]. For production purposes recombinant genes are usually transfected into the target cells as cDNA constructs in combination with a mammalian active expression cassette [5]. The gene promoter is the key element and it determines the strength and the temporal kinetics of transcription. Frequently, constitutive viral promoters are used in mammalian expression cassettes like the promoter/enhancer element of the human and mouse cytomegalovirus (CMV), the SV40 immediate early promoter or the Rous Sarcoma Virus (RSV) long-terminal-repeat (LTR) promoter [6]. However, these strong promoters can also trigger the undesired silencing phenomenon due to methylation of the promoter region and part of histones [7] or otherwise induce stress responses leading to incorrect protein folding or even apoptosis. Another problem relates to cell-cycle dependence of these promoters that may cause high cell to cell variation in the amount of recombinant protein expressed.

Due to these disadvantages, cellular promoters have been investigated for the purpose of recombinant protein expression. The best known among those are derived from house-keeping genes that encode abundantly transcribed cellular genes, such as beta-actin or ribosomal proteins. A successful example of a cell specific constitutive promoter represents that of the CHO-derived elongation factor-1 (CHEF-1) gene [8], where high expression levels were observed for several genes and different cell lines. Additional constitutive promoters that have been identified in CHO cells include the Chinese hamster Cofilin (CHCF), the CH1433e and the Chinese hamster 14-3-3 epsilon promoter. More recently the rat ferritin heavy chain (HC) gene locus has been reported by Prentice et al. [9] to be suitable for foreign gene expression.

Compared to viral promoters, little is known about mammalian promoters, since eukaryotic gene expression is more complex and requires a precise coordination of many factors for efficient gene transcription. Common to all polymerase II promoters is a shared architecture consisting of the core and a proximal promoter [10]. Some of the known core promoter elements are the TATA box, the first element identified in eukaryotic genes, the initiator element and the downstream promoter element [11]. Beside TATA-box promoters, the majority of mammalian promoters occur as so called CpG island promoters [12] where the methylation status of stretches of the CG dinucleotide and their open chromatin organization can act as a transcriptional regulator [13].

The goal of this approach is to obtain an effective endogenous CHO promoter which can be used for the expression of recombinant genes. Owing to its functional integrity described problems, observed with many viral expression cassettes, can be avoided. Here we report about the successful identification of a novel highly active gene promoter obtained from CHO cells that shows conditionally inducible gene expression at reduced temperature. In this study we investigated the full length promoter sequence and different mutant constructs in transient luciferase activity gene reporter assays.

## Results and Discussion

### 1. Identification of the CHO S100a6 gene

Potential promoter candidates were selected from a list of abundantly transcribed genes that was obtained from CHO microarray expression data [14]. The S100a6 was selected as target gene due to its high amount of mRNA levels and its response to "cold shock" conditions in various CHO parental and recombinant cell lines. For identifying the gene in its genomic context a CHO-K1 genomic lambda-phage library (Stratagene) was screened using a DIG-labeled cDNA probe specific for S100a6. Approximately 6x10^5 ^clones of the library were screened in the first round yielding only a single positive clone which was amplified during the next round and further enriched in the third screening step by duplicate plaque hybridization. The DNA of the positive lambda clone was isolated and purified. The presence, size and orientation of the genomic fragment was confirmed by PCR using gene specific primers together with the T3 and T7 promoter primer binding to the multiple cloning site of the lambda DNA. The results revealed the presence of about 6 kb of the 3' non-translated sequence of the CHO S100a6 gene, located near the T7 site in reverse direction. Amplification reactions by antisense S100a6 primers and T3 promoter primer failed, presumably due to the larger genomic region at this site. The entire insert of the lambda clone was sequenced by primer walking (GATC). In total, 14.62 kb of CHO genomic sequence were analyzed and found to contain 8.21 kb of 5' upstream sequence of the S100a6 gene encoding two other members of the S100 gene cluster (S100a4 and S100a5), and 5.15 kb of genomic CHO sequence at the 3' flanking region.

**Figure 1:**
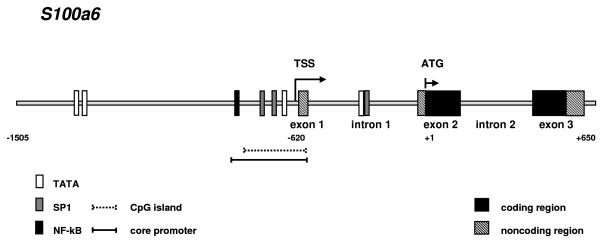
**Map of the CHO S100a6 gene**. The exon intron architecture and structural details of the CHO S100a6 gene are illustrated. Depicted sequence spans from -1505 bp of the 5' untranslated region to the end of exon 3 at +650 bp. Predicted transcriptional start site (TSS) is located in exon 1 at position -620 (arrow). Translation initiates in exon 2 after the first 24 bp at position +1. The predicted CpG island overlaps with exon 1 and extends from nucleotides -553 to -742 (dotted line). Putative transcription factor binding sites (TFBS) around TSS and in intron 1 are indicated in the figure.

To map the positions of intron-exon junctions, the CHO S100a6 genomic sequence was aligned to the corresponding locus of other mammalian species using Align and SeqMan program. The Chinese hamster S100a6 gene spans approximately 1.2 kb of chromosomal DNA and consists of three exons and two introns whereof the first exon is non-translated sequence. The organization of the CHO S100a6 gene is coherent with the human and mouse gene [15] and is illustrated in Figure 1. The initiating ATG codon could be identified in the second exon at position 25 and the translation stop signal is positioned at nucleotide (nt) 132 of exon-3. Due to the conserved exon-intron architecture (Fig. 1).a phylogenetic relationship can be assumed for this gene. CHO S100a6 coding sequence (270 bp) is 94%, 94% and 92% identical to the mouse, rat and human sequences, respectively. In contrast, the 5'-UTR, 3'-UTR and introns are predominantly divergent.

### 2. Computational analysis of functional promoter elements

Promoteractive sequences are usually located within a distance of 500 bp upstream the transcriptional start site [16]. Here, a fragment of 1.5 kb of the 5' non-translated sequence of the CHO S100a6 gene was investigated. Computational analysis of this region was performed using several freely available online prediction tools for eukaryotic Pol-II promoters (FPROM and TSSG at: http://linux1.softberry.com/berry.phtml, and a neural network based prediction program at: http://www.fruitfly.org/seq_tools/promoter.html) to identify putative TSS.

A putative core promoter was identified including a TATA box that is located at position -650 to -646. Further upstream, in close vicinity, two putative Sp1 binding sites were found at position -676 and -695. A third Sp1 consensus sequence is located at position -320 within intron 1. Prediction of the TSS was different between two programs used for analysis. The Neural Network Promoter Prediction program identified two TSS, one of which is located within intron 1 at position -420 and the second at position -620, whereas one common TSS was found at position -621 using the FPROM and TSSG program. Considering the possibility for alternative transcripts, position -621 (nt C in gtcCatcccc motif) seems to be most preferentially utilized in the CHO S100a6 gene since it is in agreement with the TSS of the human S100a6 TSS, gccCatcccc, [17]. A preference for position -620 (nt A in sequence ccAtccc) is consistent to the view that the Initiator (Inr) has the consensus element YYAN(T/A)YY (the underlined position indicates the TSS) [18].

In addition a search of potential CpG islands was performed using the cpgislands searcher http://www.uscnorris.com/cpgislands2/cpg.aspx and the EMBOSS cpgplot software http://www.ebi.ac.uk/Tools/emboss/cpgplot. One putative island region of 190 nts (GC content of >66% and an Observed/Expected ratio of 0.672) extending from nts -553 to -742 relative to the ATG start signal was predicted by the cpgislands searcher. This is in good agreement with Emboss cpg report which identifies an island of 170 nts from -578 to -747. This sequence partly overlaps exon 1 and comprises two Sp1 consensus sites (Fig. 1).

### 3. Promoter mapping and dissection

**Figure 2:**
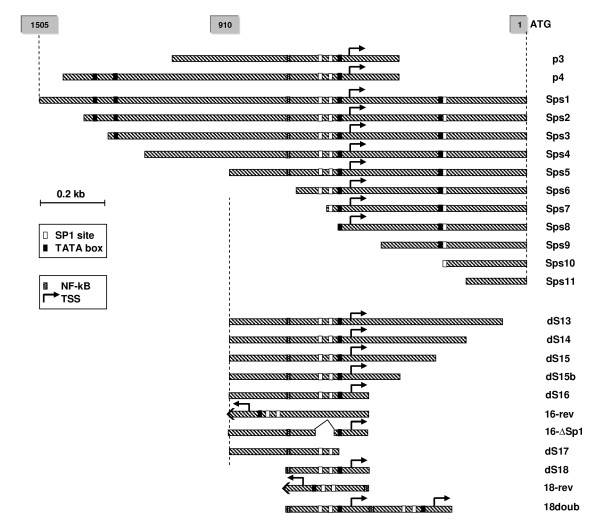
**Reporter constructs of the CHO S100a6 gene promoter**. The full-length construct (Sps1) encodes 1505 nts upstream the ATG. Grey boxes at the top indicate nts-numbers. Sps-constructs comprise the entire promoter region starting with nt 1 (unsigned) and are truncated at the 5'-side. 3' truncations (dS) start with nt 910 and are successively shortened at their 3' terminus. For double truncations (p3, p4), deletion (16-∆SP1), core promoter (dS18), tandem core promoter (18doub) and the reverse-orientated constructs see text. TFBS are indicated with boxes, TSS is marked with an arrow.

The 1.5 kb upstream region of the CHO S100a6 gene was examined for promoter activity using a transient reporter gene assay. In addition, a variety of deletion mutants were constructed by successive removal of 5' and 3' nts in order to identify transcription active regions. The different promoter constructs are shown in Fig 2.

All promoter constructs were inserted into the promoter-less basic vector pGL3-Basic (B) between the *Kpn*I-*Hind*III sites immediately upstream the reporter gene firefly luciferase. As control vector the empty pGL3-Basic plasmid was used to determine the background levels in this assay. Vector pGL3-Promoter (P) that contains the Simian virus 40 (SV40) promoter and the same vector backbone was used as positive control. Quantitative reporter assays were done in co-transfection experiments in order to standardize experimental variations like transfection efficiency or differences in cell numbers between samples. As a second plasmid pRL-SV40 which encodes the *Renilla *luciferase gene under control of the SV-40 promoter was co-transfected along with each promoter construct under study and the control plasmids. Since the obtained light signal of the *Renilla *plasmid usually is higher as compared to firefly luciferase, only one tenth of the second vector was used. For measuring the luminescence signal of both reporter proteins the Dual-Glo assay (Promega) was used which allows a consecutive measurements of both proteins from the same cell sample.

### 4. Transcriptional activity of promoter constructs in CHO cells

**Figure 3:**
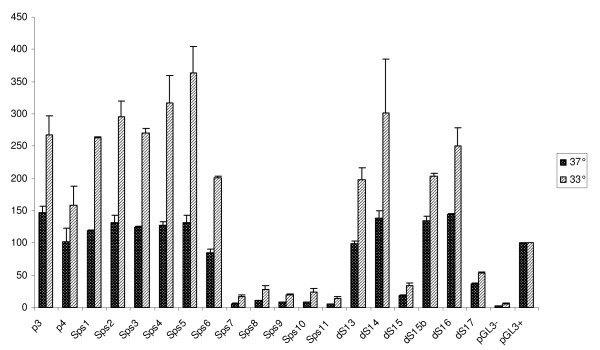
**Normalized luciferase activity of reporter constructs of the S100a6 promoter**. After co-transfections cells were incubated at 37°C and 33°C and measured for lucifearse activity after 24 hours. The average of two independent experiments with triplicate samples is shown for each construct. Relative promoter activity is indicated on the y-axis, as a percentage value of the SV40 control at the respective temperature.

The activity of 19 different promoter constructs (P3 to ds17) including the full-length variant (Sps1) together with the control plasmids was investigated for reporter gene expression. Immediately after co-transfection, cells were split and transferred to 12-well plates. One set of plates was incubated at 37°C, the other at 33°C. 24 hrs after transfection, aliquots of each sample were prepared and quantitatively analyzed. The calculation of promoter activity was done by dividing the signal from firefly luciferase by that of *Renilla *and after that the values were normalized to the corresponding pGL3-P positive control samples which were set to 100%. An overview of the activity of 19 promoter constructs plus two controls is shown in Figure 3.

The double truncation constructs, P3 and P4, are both lacking 449 nts immediately upstream the ATG translation start codon. The absence of this region does not seem to be critical for promoter functionality, since the activity was comparable to the full-length construct (Sps1) and other mutants (Sps2-Sps6) all of which include these initial 449 upstream nts at their 3' end. As concluded from P3, the removal of nts upstream 1167 from the 5' end did not reduce activity. Therefore a series of 5'-truncations were made to narrow the critical promoter region. Up to construct Sps6 which includes nts 1-763 no significant reduction was observed whereas a dramatic loss near almost background levels was noticed for the shorter constructs Sps7 to Sps11. Since construct Sps5, containing 910 nts, exhibits full activity, this 5' end was considered critical and additional constructs were designed by successively truncating 3' nts (ds13-ds17). The four long constructs dS13 - dS16 showed fairly high promoter activity (except construct dS15) and even the shortest, dS16, comprising only 332 nts reached similar expression levels compared to the full-length promoter sequence. Only dS17 which encodes 260 nts fell below 30 percent of the top constructs and below 50 percent of the SV40 expression value. This clearly indicates that missing nts between dS17 and dS16 are important for promoter activity although their absence does not completely diminish the functionality. A closer look at this sequence reveals the presence of the predicted CpG island (-747 to -577) that overlaps the predicted TSS and a TATA-box occurring in this region. Constructs dS16 and dS17 were designed to investigate the effect of regulatory elements. Common to both constructs is the presence of two SP1 transcription factor binding sites (TFBS) at positions 671-676 and 690-695, but dS17 lacks the TATA-box and the predicted TSS at 620/621. This may explain the drop in activity observed in the shortest construct. Further we investigated the effect on transcription by deleting one of the SP1 sites. Construct Sps6 which encodes nts 1 to 763 including both SP1 sites, showed 80-90% expression strength of the control vector. In contrast, Sps7 which encodes only nts 1 to 683 and lacks one essential SP1 binding side, completely lost promoter functionality. According to these results the core promoter sequence including both TFBS and the predicted transcription start site is located in exon-1 which overlaps with the putative CpG-island.

Promoter analyses under hypothermic conditions revealed even higher activity for all tested variants. In general, reduced temperature caused a boost in expression of about 200% on average (Figure 3). At 33°C, three of the 5' deletion constructs Sps4, Sps5 and Sps6 showed a substantial enhancement in reporter gene expression of more than 2-fold when compared to the corresponding samples of 37°C and more than 3-fold of the SV40 control vector under temperature shift conditions. Sps4 and Sps5 showed the strongest response to temperature change and the other 5' truncation constructs, Sps1, Sps2 and Sps3 could be activated >2-fold upon a shift to 33°C.

With length decreasing a reduction in temperature response was observed; see dS13-dS16 and P3 and P4. These mutants lack up to 578 nts in the 3' region which seems to contribute to this lower temperature sensitivity since expression levels were found to range between 1.5 to 1.8-fold of the control values at 37°C under these experimental conditions. We observed that reporter gene expression of all promoter variants is strictly time dependent and rapidly increasing over time. Whether this is caused by the accumulation of the reporter protein in the cell remains to be examined. Consequently, all measurements were done exactly 24 hours after transfection of the cells. Remarkably, the ratio of expression levels between the SV40 control and the constructs under study stayed constant up to a sampling time of 48 hours in the temperature shift experiments.

### 5. The core promoter sequence

**Figure 4:**
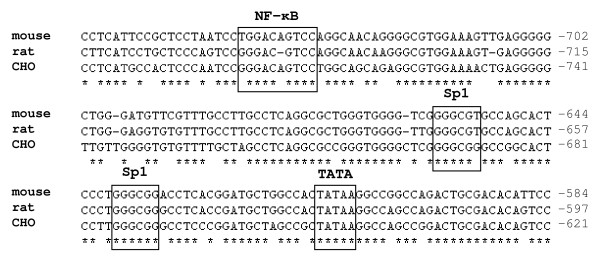
**Regulatory elements in the S100a6 promoter region**.  Alignment among mouse, rat and Chinese hamster (CHO). NF-ĸB, SP1 binding sites and TATA box are conserved sequences and indicated by boxes.

Our next aim was to further minimize the promoter and characterize more precisely its functional elements. Concluding from our results we identified sequence elements upstream 579 (e.g. dS16: 579-910) essential for full promoter activity. Truncations at the 5'-site as far as position 910 (dS13-dS16, Sps5) did not result in diminished activity. Even in construct Sps6, with all nucleotides deleted upstream position 763, indeed a decrease but no loss of expression was detected resulting in a slightly lower activity than the SV40 control. According to these findings, we designed a new "minimal" construct, dS18, which extends from nts 579 to 800. The 5'-end position 800 was chosen, since a putative NF-ĸB binding site (771-780) was included. This site is conserved among Chinese hamster, mouse and rat (Figure 4) and its involvement in the regulation of the human S100A6 gene expression was studied by Joo et al. [19]. Also the two SP1 sites and the TATA box motifs are present in the promoter region of the other two species.

**Figure 5:**
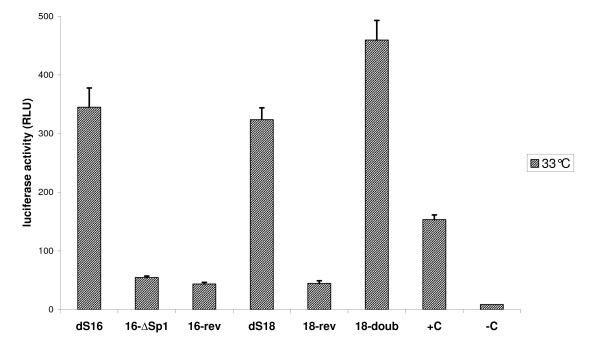
**Luciferase activity of S100a6 promoter constructs**. 5 different constructs of the S100a6 promoter in series 4 compared to dS16, +C (pGL3-P, the positive control) and -C (pGL3-B, the negative control). Each construct was co-transfected with pRL-SV40 into CHO cells. Cell suspensions were measured for luciferase activity 24 hours after transfection and incubation at 33°C. All relative luciferase activities were normalized to Renilla luciferase activity. The average of two independent experiments with triplicate samples is shown for each construct.

In our study we directly compared dS18 (222 nts) and dS16 (332), the so far smallest construct with full promoter activity. Its activity was found equal to dS16, the initially identified minimal promoter. The results of this paragraph are summarized in Figure 5. The NF-ĸB binding site seems to be necessary for full promoter function since lacking of this site in Sps6 (1-763) results in decreased activity down to a level of 65% as compared to Sps5 (1-910).

Several cis-acting elements can also be important for reverse-oriented transcription except for the original TATA box. The GC box has been reported to exhibit such reverse function and some mammalian promoters have been identified to show antisense activity, e. g. murine *Dfna5 *[20]. Thus the function of S100a6 core promoter in inverse orientation was also tested. We designed the inverse constructs dS18-rev and dS16-rev to determine if transcription can be initiated in this configuration. Both showed low but detectable activity of about 20% of theirs original sequence. This activity was similar to the level of the SP1 deletion construct 16-∆SP1 (see below).

In order the study the function of SP1 sites, both were deleted from construct dS16 generating 16-∆SP1 (579-658, 721-910) leaving only the TATA-box intact. Lacking of only 64 bp, including two putative SP1 sites, dramatically decreased promoter function to about 20% indicating that both conserved SP1 sites are essential for functioning of the S100a6 promoter. This observation is relevant to the view that the SP1 site, a GC hexanucleotide, is sufficient for SV40 promoter activity and frequently appears as multiple copies.

Several studies have investigated the effect of promoter duplication on homologous and heterologous gene expression. It was shown that a second copy of the promoter or at least its essential regions can function like cis-acting enhancer elements. This strategy has been evolutionally developed by eukaryotic viruses in order to enhance the synthesis of their RNA genome [21]. For biotechnological applications it is important to achieve high and reliable expression levels. In an attempt to enhance transgene expression, the effect of adding extra TFBS by duplicating parts of the promoter region while maintaining the spatial arrangement was analysed by Pena and Whitelaw [22]. For the purpose of such a use we generated construct 18-doub, having two consecutively repeated core promoter sequences (2 × 222 bp) to test for increased activity. We observed that this tandem repeat stimulates transcription more effectively (1.4 fold of dS18) than a single element, and the distance of the SP1 site(s) from the TATA box may have some influence.

### 6. Conformation of temperature dependent promoter activity by quantitative real-time (qRT) PCR

**Figure 6:**
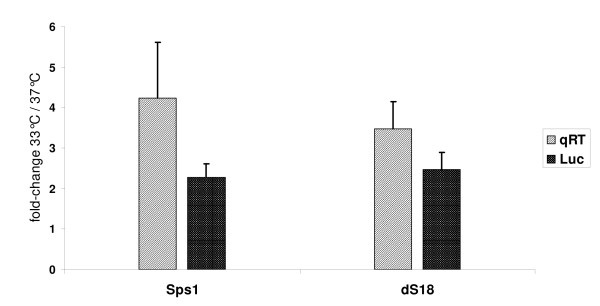
**Temperature-shift induced change in gene expression. **Two representative promoter constructs, the Sps1 full-length and the dS18 core promoter sequence, were analyzed for relative mRNA levels (qRT) and luciferase protein expression (Luc). Analyses were performed at two different temperatures at 33°C and 37°C measured after 24 hours. The shown values represent the mRNA and protein ratios at 33°C relative to 37°C (y-axis) normalized to pGL3 control vector.

Quantitative real-time PCR was used to verify the results obtained by luciferase reporter gene expression studies of different constructs in temperature shift experiments. Measured protein levels at standard (37°C) and reduced temperature (33°C) were compared to specific mRNA levels of two representative promoter constructs, namely the full-length construct (Sps1) encoding 1505 nts immediately upstream the ATG and the 222 nts core promoter sequence (dS18). The same constructs were assayed in parallel for Luciferase protein expression at both temperatures. Figure 6 depicts the fold-change values of mRNA and protein levels at 33°C compared to 37°C for the two promoter variants at 24 hours post transfection.

### 7. The role of mRNA stability in regard to transcript abundance of the S100a6 gene at low temperature

Insearch of a possible mechanism for enhanced mRNA levels upon lowering temperature we compared the structural and functional properties of the S100a6 gene to well characterized mammalian cold shock proteins (CSPs), namely the cold-inducible RNA binding protein (CIRP) [23] and RNA binding motif protein 3 (Rbm3) [24]. They have been recognized as regulators of gene expression by acting as RNA chaperones through binding of misfolded RNA thereby preventing transcription termination [25]. Their expression is modulated by alternative mRNAs with IRES-like activity that increases translation [26]. The 5'-UTR of CHO S100a6 gene was therefore searched by a web-based IRES secondary structure prediction tool [27]http://polya.umdnj.edu/polya_svm, but no IRES-like structure could be predicted, whereas three potential cap-independent translation initiation sites where found in the 5'-UTR of the human CIRP gene (GenBank: D78134) that served as control. Thus, the CHO S100a6 gene does not seem to have structural similarity to the CSP's 5'-UTR and we assume another regulatory mechanism. Another structural feature of CSPs is the presence of widely conserved binding domains (anti-parallel ß-strands) that accounts for affinity to single stranded RNA and DNA. Appar.ntly, the S100a6 protein lacks such motifs as well as the ability to attach to RNA/DNA. It rather interacts with many biological targets via protein-protein binding.

In addition, we did a comparative literature search for the regulatory and functional features of the human S100a6 ortholog with a focus on the gene promoter and various stimuli that have been identified to upregulate its expression. These include growth factors, inflammatory cytokines, hormones and tumor inducing agents [28]. Upon stress conditions such as ischemia, mechanical force, irradiation or oxidative stress the S100A6 gene promoter is activated at the transcriptional level by upstream stimulatory factor (USF) and NF-ĸB [29]. USF binds to the E-box response element, a hexanucleotide motif that is also present in the CHO S100a6 gene promoter at almost the same position: E-box CHO: -273/-268; human: -283/-278 upstream the transcription start site. Recent data provide evidence that USF may be another stress responsive transcription factor activated by UV and osmotic shock, heat-shock and cadmium [30].

**Figure 7:**
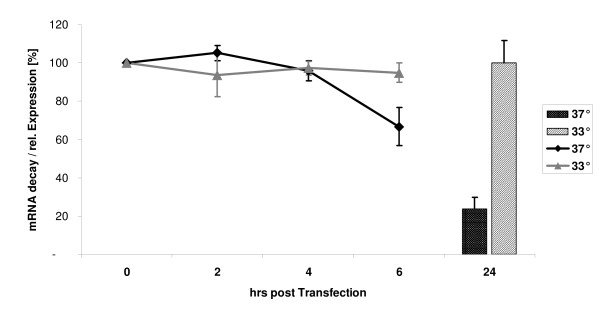
**mRNA stability of cold shock transcripts**. The full-length promoter construct (Sps1, 1505 nts) was analyzed by quantitative RT-PCR after inhibition of de novo transcription in order to determine transcript stability at 33°C and 37°C. The y-axis shows the percent-values of the amount of mRNA of the Luc reporter construct at indicated time points (hours after transfection). Time zero is set to 100%. After 6 hrs at 37°C the mRNA decline was 33% as compared to 5% at 33°C. The columns show the ratio of transcript abundance (33°C-value is set to 100%) that was detected at 24 hrs after transfection at both temperatures.

Since upregulation of the S100a6 gene, induced by stress or other stimuli, is primarily based on transcriptional control we conclude that cold shock stress in our experiments predominantly triggers a transcriptional response. To test as to whether RNA turnover is also involved in regulation we measured mRNA stability of the promoter constructs at lower (33°C) and standard culture temperature. For that purpose the full-length promoter construct (Sps1) was selected. The transcription inhibitor actinomycin D was added to the culture and the level of the target mRNA was determined at several time points by quantitative RT-PCR. This allows calculating mRNA decay and the corresponding half-live. As internal control β-actin was selected as reference since its mRNA expression is considered not to be affected by lowering culture temperature [31]. Due to the short lived nature of mRNA we followed its concentration during 6 hours after inhibiting transcription. At each time point mRNA levels of the reporter gene ß-actin stayed remarkably constant. Also, mRNA levels of the promoter construct did not differ significantly after the first 4 hours at both temperatures. However, 6 hours after actinomycin D treatment a decay of about 33% was detected at 37°C, whereas in the parallel test culture at 33°C mRNA only decreased about 5% (Figure 7). This observation indicates that mRNA stability is enhanced by lowering culture temperature which also contributes to higher mRNA levels.

## Conclusions

The identification of the CHO S100a6 promoter sequence was based on the analysis of microarray transcriptomics data described by [14]. Due to sparse genomic resources that are publicly available for the Chinese hamster we took a conventional approach for target identification by screening a CHO-K1 genomic lambda phage library. The isolated positive phage clone comprises a fragment of 14,6 kb genomic CHO DNA encoding the S100 gene locus including family members a4, a5 and a6. The CHO S100a6 gene consists of three exons and two introns, and its coding sequence is highly conserved between mouse, rat and human. According to computational analysis, a sequence region of 1,505 bp upstream of ATG start codon was termed as full-length promoter and tested together with a number of promoter variants in functional promoter assays. A core promoter region of only 222 bp could be identified revealing almost similar activity than the full-length construct. Several regulatory elements were identified as essential factors to allow for full promoter function. A drop in activity was observed when either the TATA-box or the NF-ĸB binding site was absent. Also the lack of two SP1 binding sites in the core promoter region caused a drastic reduction in activity. On the other hand, functional activity of the "minimal" promoter could be considerably enhanced when two tandem repeats were joined together. Considering additional aspects beside transcriptional upregulation of the S100a6 gene we could also identify mRNA stability that contributes to transcript abundance at lower temperature.

In addition, the CHO S100a6 promoter shows conditionally inducible gene expression at reduced temperature resulting in two- to three-fold increase in gene expression compared to basal activity at standard temperature. This temperature dependent activity can be advantageous to reduce expression during the initial growth phase and at later production phase it allows to boost expression of the recombinant gene. It is particularly valuable that this increase of productivity is achieved at low temperature culturing conditions which normally reduces overall cell metabolism of competitive processes but obviously not the expression of the gene of interest. Another benefit is the potential for the manufacturing of toxic products since gene expression and cell metabolism do not negatively interfere as compared to conventional mammalian production technology.

The area of improving recombinant protein production by the use of reduced temperature cultivation has been documented by several investigators [32,38]. Although lowering the culture temperature often increases specific productivity, its effect on productivity is variable among CHO cell lines [39] and there are conflicting reports in the literature. Al-Fageeh et al. [37] suggested optimization of cell growth at 37°C followed by a "cold-shock" but contradictorily it has been demonstrated that adaptation of CHO cells to low culture temperatures can enhance specific growth rates whereas recombinant protein production decreases [40].

In case of the S100a6 promoter, increased activity after "cold shock" treatment does not result from prolonged generation time like in batch cultures or effects that can be attributed to reduced cell metabolism since we determined expression levels shortly after transient transfections. Higher productivity is directly connected to increased specific mRNA transcription rate which we observed for parental and recombinant cell lines, including also CHO-K1. The use of this promoter for high level protein expression under hypothermic condition therefore demonstrates an attractive alternative in production techniques since it combines both, a novel genetic construct that is directly linked to the individual process conditions with the potential for improving the performance also for other mammalian expression systems.

## Methods

### Cell culture, growth media and cultivation

Dihydrofolate-reductase deficient CHO cells (ATCC, Rockville, MD, USA) were cultured in Dulbecco's modified eagle's medium (DMEM) and Ham's F-12 medium mixed 1:1, supplemented with 4 mM L-glutamine, 0.1% (w/v) pluronic, 0.25% (w/v) soya-peptone, 13.60 mg/l of hypoxanthine and 3.88 mg/l of thymidine, at 37°C under 7% CO_2_. The cells were routinely passaged twice a week (1:6) at a starting density of 1 × 10^5 ^cells/ml in a spinner flask and used for transient transfection assays at the amount of 4 × 10^6 ^cells per sample.

### Screening of a CHO genomic lambda-phage library

A CHO-K1 genomic library in the Lambda FIX II vector (Stratagene Inc, La Jolla, Calif) was screened with a probe specific for the Chinese hamster S100a6 gene. cDNA was synthesized with SuperScript^® ^III Reverse transcriptase (Invitrogen) in a volume of 20 μl and 250 ng of CHO total RNA at 42°C for 1 h. 2 μl of the reaction product were further amplified by PCR at 98°C for 30 s and 34 cycles of 98°C for 10 s, 60°C for 30 s and 72°C for 15 s followed by a final extension at 72°C for 5 min using Phusion DNA polymerase (Finnzymes) and primers S100B (5'-ATGGCATGCCCCCTGGATCAG-3') and S100F (5'-CATTGTAGATCAAAGCCAAGG-3'), respectively. 100 pg of the amplified RT-PCR product were labeled with DIG-dUTP in a final volume of 50 μl using PCR DIG Probe Synthesis Kit, according to the DIG application manual (Roche). All cloning procedures were performed as described by Sambrook and Russell 2001 [41], restriction enzymes and other modifying enzymes were purchased from New England Biolabs (NEB, USA) and used according to the manufacturer's recommendations.

### Plaque lifts

For one dish (145 mm) that was used for screening and plaque hybridization, fresh *Escherichia coli *XL1-Blue MRA (P2) (600 μl of OD600 = 0.5 cells diluted with 10 mM MgSO_4_) were infected with 10 μl of 50,000 plaque-forming units for 20 minutes at 37°C. Then 7 ml of NZY top agar at ~50°C were added, mixed and spread onto a prewarmed (37°C) NZY agar plate. The plaques became visible after 8 hours incubation at 37°C. After plaque formation, the culture dishes were stored at least 2 hours at 4°C, blotted on nylon membrane (Roche), denatured and neutralized. The step from preparing the plating cultures and performing the plaque lifts were done according to the instruction manual for Lambda FIX II Library (Stratagene).

### Hybridization and detection

Before hybridization, the DNA was fixed to the nylon membrane by baking at 80°C for 2 hours. Pre-hybridization (39°C for 1 hour) and hybridization (39°C for overnight) were performed in the same solution, standard buffer (5x SSC, 0.1% (w/v) N-lauroylsarcosine, 0.02% (w/v) SDS and 1% blocking reagent). 10 ml buffer containing 10 μl of DIG-labeled probe were used for hybridization for one nylon membrane. Membranes were washed twice with low stringency wash buffer (2x SSC, 0.1% SDS) at room temperature followed by high stringency wash buffer (0.5x SSC, 0.1% SDS) at 65°C. After washing and blocking, the antibody solution (1:10,000 diluted Anti-Digoxigenin-AP (Roche) in blocking solution) was added to the membrane. Membranes were washed, equilibrated, covered with CDP-Star (NEB) and exposed to Lumi-Imager (Roche) to detect with a chemiluminescent assay. All steps after performing the plaque lifts were done according to the DIG application manual (Roche). A square area of agar corresponding to the location of a single positive clone was picked from the plate and confirmed by a second and third screening step. Purification of the lambda DNA was done with a Lambda kit (Qiagen Inc). The DNA was used in PCR reactions with T3 (5'-AATTAACCCTCACTAAAGG-3') and T7 (5'-TAATACGACTCACTATAGGG-3') and S100a6 gene specific primers A-F for veryfing the presence and location of the gene.

(primer name, sequence (5'-3'), strand/location).

A CTCCTTTGGCTCTTCGCTGTC sense/exon 1

B ATGGCATGCCCCCTGGATCAG sense/exon 2

C CCTTCTTGTGGCCATCTTCC sense/exon 2

D CTGAGATTGCAAGGCTGATGG sense/exon 3

E GCCAATGGTGAGCTCCTTCTG reverse/exon 2

F CATTGTAGATCAAAGCCAAGG reverse/exon 3

The lambda DNA was sequenced by GATC Biotech (Germany).

### Generation of promoter constructs

5'- truncation constructs: according to the sequence information of the lambda DNA, several 5' deletion constructs were generated by PCR with the use of reverse primer (-1)F GATGATAAGCTTAATTGACCACTGGGCTAGAAG together with 1 of 11 sense primers as listed in below:

(primer name, sequence 5'-3'):

(-1505)B GATGATGGTACCGCATGCTGGCTGGGCTGGG

(-1343)B GATGATGGTACCTGAGACAGGGTTTTATATAGCC

(-1294)B GATGATGGTACCGGATCAACCTACTGAGCTATAT

(-1206)B GATGATGGTACCCACAAGTATTTACACTGAGATTC

(-910)B GATGATGGTACCATGCCGGAGTCACGAGTCAC

(-763)B GATGATGGTACCAGAGGCGTGGAAAACTGAGG

(-683)B GATGATGGTACCACTCCTTGGGCGGGCCTC

(-663)B GATGATGGTACCGGATGCTAGCCGCTATAAGG

(-534)B GATGATGGTACCAGGTCGGCTCCTGGGCTGG

(-322)B GATGATGGTACCTAGGGCGGCTCCCCGAGT

(-256)B GATGATGGTACCTCGCAGTGTGTGGTCCTGTC

The resulting constructs were designated Sps1 to Sps11, starting with the full-length construct derived with primer (-1505)B to the smallest obtained with primer (-256)B.

3'- truncation constructs: Constructs were generated by PCR with the use of sense primer (-910)B GATGATGGTACCATGCCGGAGTCACGAGTCAC together with one of the following reverse primers (name, sequence 5'-3'):

S-HINDIII(-115)-F GATGATAAGCTTTCAGGGATGTAAGAACGGAAGC

S-HINDIII(-231)-F GATGATAAGCTTCTTCCTGACAGGACCACACAC

S-HINDIII(-344)-F GATGATAAGCTTGCTTGCCTGGCACAACCAAGC

S-HINDIII(-461)-F GATGATAAGCTTTAGACCACCCGCGGAACCCG

S-HINDIII(-579)-F GATGATAAGCTTGGCAGGTAGACAGCGAAGAGC

S-HINDIII(-651)-F GATGATAAGCTTGCGGCTAGCATCCGGGAGG

The resulting constructs were designated from dS13 to dS17 according to the series of primers from (-115)F to (-651)F.

5' and 3' double truncation constructs: fragments P3 and P4 were amplified by PCR using primers: -1425B (5'-GATGATGGTACCGGAAGAAGCGAGTTAGACAG-3') and -1167B (5'-GATGATGGTACCTACGGTGTGAAGCAGCAGTG-3') together with the reverse primer -450F (5'-GATGATAAGCTTAGACCCCAGTGTAGACCACC-3').

deletion construct: a 64 bp GC-rich fragment located at positions -722 to -658 of the S100a6 promoter (5'CTAGCCTCAGGCGCCGGGTGGGGCTCGGGGCGGGCCGGCACTCCTTGGGCGGGCCTCCCGGATG-3'), containing two putative SP1 binding sites, was removed from construct dS16 by *Nhe*I digestion. 4 μg of construct dS16 DNA were digested with 20 U of *Nhe*I, purified by a GFX kit and subsequently religated with T4 DNA ligase, yielding construct 16-∆SP1

reverse-orientated, minimal and double promoter constructs: To generate the minimal, reverse and double promoter constructs primers listed below were used (primer name, sequence 5'-3'):

HINDIII(-910)B GATGATAAGCTTATGCCGGAGTCACGAGTCAC

KPNI(-579)F GATGATGGTACCGGCAGGTAGACAGCGAAGAGC

KPNI(-800)B GATGATGGTACCCCTCATGCCACTCCCAATCC

HINDIII(-800)B GATGATAAGCTTCCTCATGCCACTCCCAATCC

XHOI(-791) B GATGATCTCGAGACTCCCAATCCGGGACAGTC

XHOI(-589) F GATGATCTCGAGCAGCGAAGAGCCAAAGGAGTG

A reverse-oriented construct of dS16 (16-rev) was generated by cloning of the fragment amplified with the use of primers *Hind*III(-910)B and *Kpn*I(-579)F into the pGL3-Basic vector. The minimal promoter construct, dS18, was generated by removing a 110 bp upstream region from dS16, using primers *Kpn*I(-800)B and (-579)F for PCR amplification. Construct 18-rev was generated by PCR with primers HindIII(-800)B and KpnI(-579)F, followed by cloning into the vector in reverse orientation. Construct 18double, consisting of two tandem repeats of the core promoter sequence (dS18), was generated by amplifying two copies of the core sequence, one with *Kpn*I(-800)B and *Xho*I(-589)F and the second with *Xho*I(-791)B and (-579)F. The products of these PCRs were cut with corresponding enzymes and subsequently ligated together.

All fragments were cloned into pGL3-Basic at *Kpn*I-*Hind*III sites and sequenced.

The PCR reactions for the constructs were performed in a reaction volume of 25 μl containing 1 ng of lambda DNA as template, 0.2 mM of each dNTPs, 0.4 μM of each primer and 1 unit of KOD Hifi DNA polymerase (Novagen) at the following cycling conditions: 95°C 2 min, 30 cycles 98°C 15 s, 58°C 5s and 72°C 20s, final extension at 72°C 5 min. Amplifications for constructs P3 and P4 were performed in 50 μl containing the same amount of template DNA and dNTPs as above, 0.5 μM of each primer and 1 unit of Phusion DNA polymerase (Finnzymes). PCR cycles were: 98°C 2 min, 30 cycles of 98°C 10 s, 60°C 15 s and 72°C 45 s and a final extension at 72°C for 7 min.

### Transient transfection assay

The constructs were co-transfected along with the Renilla luciferase reporter vector pRL-SV40 into CHO dhfr^- ^by using Amaxa's Necleofector (Lonza). 4 × 10^6 ^cells were transferred to a 15 ml tube and centrifuged at 1000 rpm 10 min and resuspended with the nucleofection mixture containing 10 μg of plasmid DNA, 1 μg of pRL-SV40 in 100 μl of NucleofectorTM solution and subjected to electroporation. Subsequently, the cells were transferred into a pre-warmed culture medium in 6-well plates and incubated at 33°C and 37°C. The luciferase activities were measured on a Biotek Synergy 2 luminometer (Gen5 software) by using the Dual-Luciferase Reporter Assay System (Promega) preferentially after 24 hours. To normalize for transfection efficiency, the promoter activity of each construct was expressed as the ratio of firefly luciferase activity relative to Renilla luciferase activity. pGL3-Basic and pGL3-Promoter were used as negative and positive controls, respectively.

### Quantitative real-time (qRT) PCR

Total RNA was extracted from 4 × 10^6 ^cells transfected with the promoter constructs, using the TRIZOL reagent, following the instructions for RNA isolation (Invitrogen TM, Life technologies). The yield and purity of the isolated RNA were determined by measuring the absorbance at 230, 260 and 280 nm using an ND-1000 spectrophotometer (NanoDrop Technologies, DE). The Eukaryote total RNA Nano assay together with Agilent's 2100 bioanalyzer were used for quality control of the isolated RNA according to the RNA 6000 Nano LabChip kit (5065-4476) standard protocol. First-strand cDNA synthesis of 1.5 μg total RNA was performed with the SuperScript III Reverse Transcriptase kit (Invitrogen). The synthesized cDNA served as template for quantitative real-time PCR reactions (1 cycle at 95°C, 2 min; 40 cycles at 95°C, 15 s; 60°C, 60 s) with the Rotor-Gene Q Real-Time PCR cycler (Quiagen). GoTaq qPCR Master Mix (Promega) was used in combination with the primers listed below and the corresponding cDNA template. For relative quantification of the mRNA levels of the samples the Rotor gene 6000 software and the 2^-^ΔΔC T comparative method [42] were used.

Luc135_se ACATATCGAGGTGGACATCAC

Luc199_as TAGCTTCTGCCAACCGAACG

n-Actb-back TTGACTCAGGATTTAAAAACTGG

n-Actb-for TGCTCCAACCAACTGCTGTCG

### Measurement of mRNA stability

The mRNA stability of the Luciferase reporter gene normalized to the β-actin reference gene was determined via quantitative real-time PCR after blocking of new RNA synthesis by addition of actinomycin D at a final concentration of 5 μg/ml. Following actinomycin D treatment, 4 × 10^6 ^cells were harvested at designated time points (0 h, 2 h, 4 h, and 6 h) and total RNA was prepared and reverse transcribed into cDNA as described in section "Quantitative real-time (qRT) PCR". The fold change in Luc mRNA level is indicated as percentage mRNA decay. The mRNA level at time point zero (before treatment with actinomycin D) was considered as 100%.

## Authors' contributions

HT carried out the molecular biology work, the cell culture and transfection experiments and helped to draft and write the manuscript. MB was in charge of the quantitative real-time PCR assays, cell culture experiments and analytics. HT and MB contributed equally to this study. JP performed the luciferase reporter assays and participated in computational analysis of promoter sequences. FH participated in conception of the study and in the design of experiments. WE conceived the study, was responsible for study design and coordinating and writing the manuscript. All authors read and approved the final manuscript.
